# Role of Hypofractionated Stereotactic Radiosurgery in Recurrent Pineal Parenchymal Tumors of Intermediate Differentiation: A Case Report and Review of the Literature

**DOI:** 10.7759/cureus.9709

**Published:** 2020-08-13

**Authors:** Caglayan Selenge Beduk Esen, Gozde Yazici, Mustafa Berker, Faruk Zorlu

**Affiliations:** 1 Radiation Oncology, Hacettepe University Medical School, Ankara, TUR; 2 Neurosurgery, Hacettepe University Medical School, Ankara, TUR

**Keywords:** recurrent, pineal parenchymal tumor of intermediate differentiation, cyberknife radiosurgery, image-guided robotic radiosurgery, frameless

## Abstract

A pineal parenchymal tumor of intermediate differentiation (PPID) is a rare entity, and optimal treatment is still unclear. Combined multimodality treatment should be considered in PPID due to high recurrence rates. Gross total resection is the first choice of treatment, however, it may not be feasible in every case due to location. Stereotactic radiosurgery (SRS) can be considered for the treatment of primary and recurrent disease, as it enables us to deliver a high radiation dose to the target while minimizing radiation exposure to normal tissue. In this report, we present a case treated with hypofractionated SRS for recurrent/metastatic PPID after the primary tumor was controlled with the combination of surgery and conventionally fractionated radiotherapy.

## Introduction

Pineal gland tumors are rare, constituting less than 1% of all primary central nervous system (CNS) tumors [1]. Pineal parenchymal tumors (PPT) are the second most common tumors of the pineal region after germ cell tumors and account for approximately 14%-27% of all pineal region tumors [1].

Due to its rarity, there is no standard treatment for pineal parenchymal tumors of intermediate differentiation (PPID). Gross total resection is an important prognostic factor for pineocytoma and low-grade PPID, however, multimodality treatment, such as surgery, radiotherapy (RT), and chemotherapy, should be considered in high-grade PPID and pineoblastomas, as they have a high risk of recurrence [2-3].

The surgery may not be feasible in all cases due to the tumors’ proximity to neurovascular structures. Although microsurgical approaches have reduced the morbidity of open surgical procedures, potential adverse effects may still be observed. Stereotactic radiosurgery (SRS) may be an alternative to surgery due to its advantage of delivering high radiation doses to a small focused target while minimizing the normal tissue doses. We present a 59-year-old woman who has been treated with intensity-modulated radiation therapy (IMRT) for primary PPID and hypofractionated SRS for recurrent metastatic lesions.

## Case presentation

**Figure 1:**
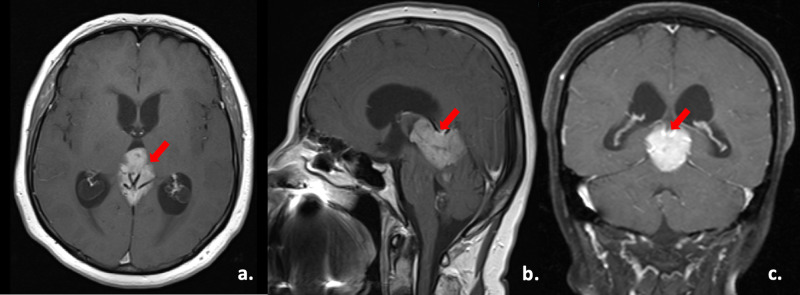
Magnetic resonance images of the mass a, b and c: Axial, sagittal, and coronal contrast-enhanced magnetic resonance imaging (MRI) scans of the mass originating from the pineal region (red arrows), respectively.

A 59-year-old woman presented with a seizure in 2012. Her medical history was only remarkable for hypertension. Gadolinium-enhanced magnetic resonance imaging (MRI) showed a 4.2x3.2x3.8 cm sized mass originating from the pineal region, associated with obstructive hydrocephalus (Figure 1). 

The mass was resected totally and the histopathological examination revealed a hypercellular tumor composed of tumor cells with narrow cytoplasm without necrosis, and the neoplasm had rosette-like structures. The neoplastic cells were immunohistochemically positive for synaptophysin and neurofilament protein but negative for glial fibrillary acidic protein (GFAP). The Ki-67 index was 3.5%-4%, and the mitotic count was 5-6 per 10 high-power fields. Pathological findings were consistent with PPID (World Health Organization (WHO) grades II-III) [4]. The patient received adjuvant radiotherapy to a total dose of 56 Gy in 28 daily fractions with IMRT. The postoperative tumor bed with a 1 cm radial margin was defined as the clinical target volume (CTV), and a 5 mm margin was defined around the CTV for the delineation of the planning target volume (PTV). She did not receive any chemotherapy. The patient was followed up with a cranial MRI every six months for the first two years and then yearly.

**Figure 2:**
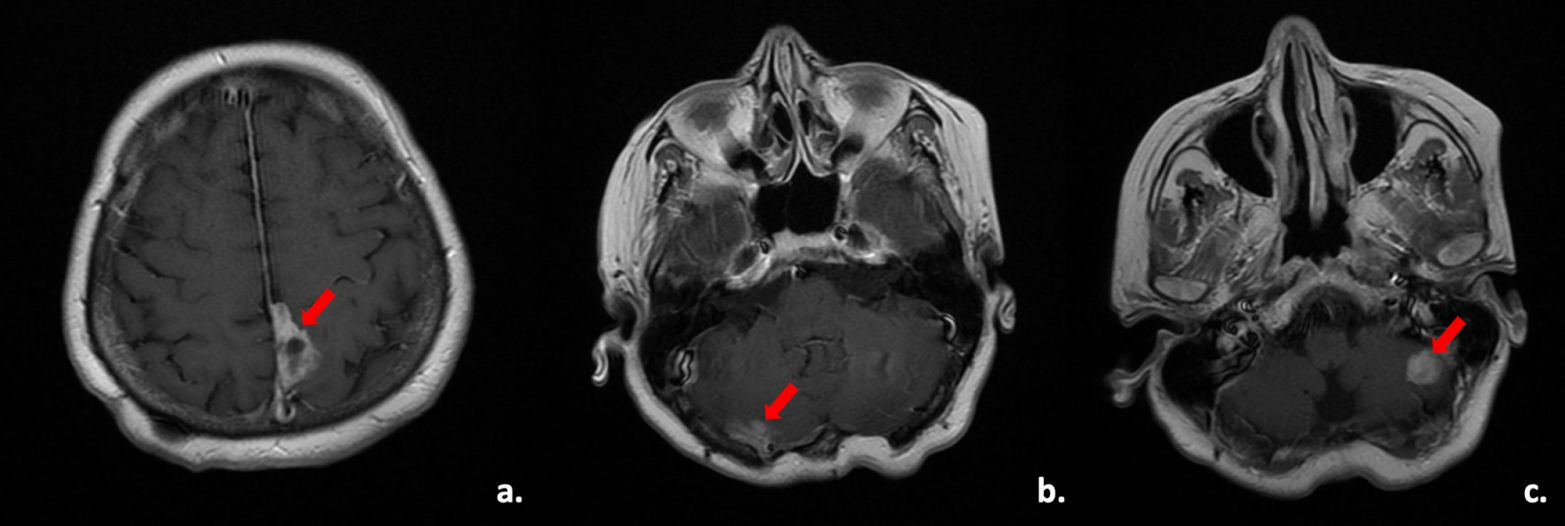
MRI scans of the lesions a. Axial contrast-enhanced magnetic resonance imaging (MRI) scan of the lesion in the left parietal lobe (right arrow). b and c. Axial contrast-enhanced MRI scan of the lesions in right and left cerebellar hemispheres (red arrows), respectively.

In April 2018, the follow-up brain MRI showed a 2.8x2.1 cm sized mass in the left parietal lobe, 1.8x1 cm and 1.3x1 cm sized masses in the right and left cerebellar hemispheres, respectively, and all were located extra-axially (Figure 2).

**Figure 3:**
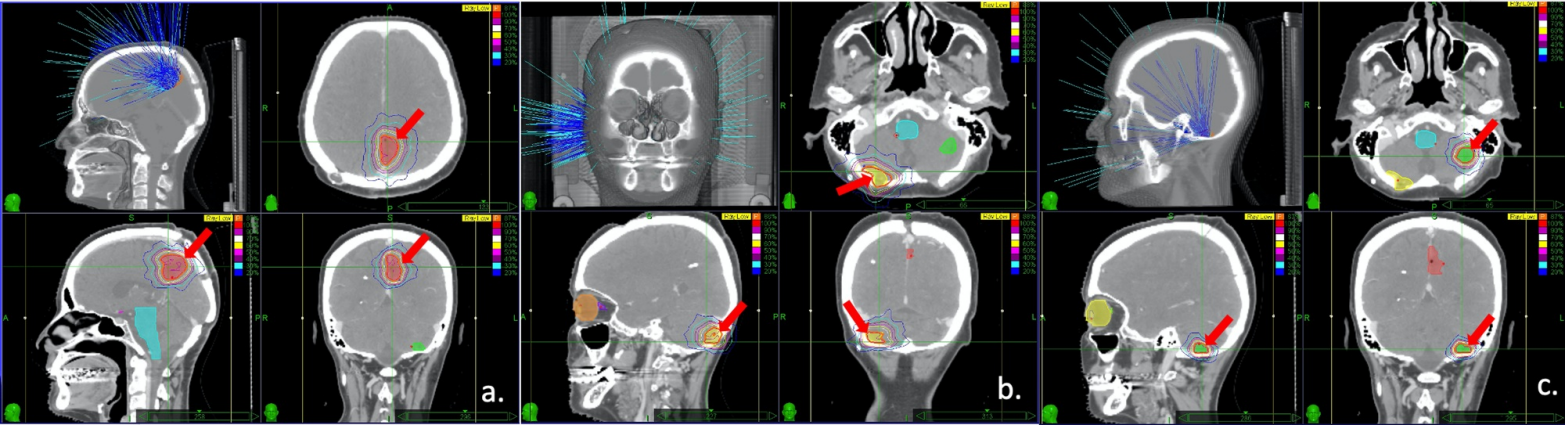
Treatment planning images a. Axial, sagittal, and coronal views of the treatment planning computed tomography (CT) scan of the resection cavity in the left parietal lobe. The orange line is the prescribed isodose (87%). b. Axial, sagittal, and coronal views of the treatment planning CT scan of the lesion in the right cerebellar hemisphere. The orange line is the prescribed isodose (88%). c. Axial, sagittal, and coronal views of the treatment planning CT scan of the lesion in the left cerebellar hemisphere. The orange line is the prescribed isodose (87%).

The spinal MRI showed millimetric pial enhancements and thickened dura at the cervicomedullary and thoracolumbar junctions and cauda equina. Computed tomography (CT) scans of the thorax and the abdomen and the mammogram did not show any lesion suggesting a second primary tumor. The mass in the left parietal lobe was resected totally and the pathological findings were consistent with PPID (WHO grades II-III). The Ki-67 index was 10% and the mitotic count was 6 per 10 high-power fields. Since the patient had leptomeningeal involvement of the spine, we performed craniospinal irradiation to a total dose of 30.6 Gy in 18 fractions. We then performed hypofractionated SRS boosts to cranial lesions (the left and the right cerebellar lesion, and the resection cavity in the left parietal lobe) using Cyberknife® (Accuray Incorporated, Sunnyvale, California) to a total dose of 25 Gy in five fractions every other day (Figure 3).

**Figure 4:**
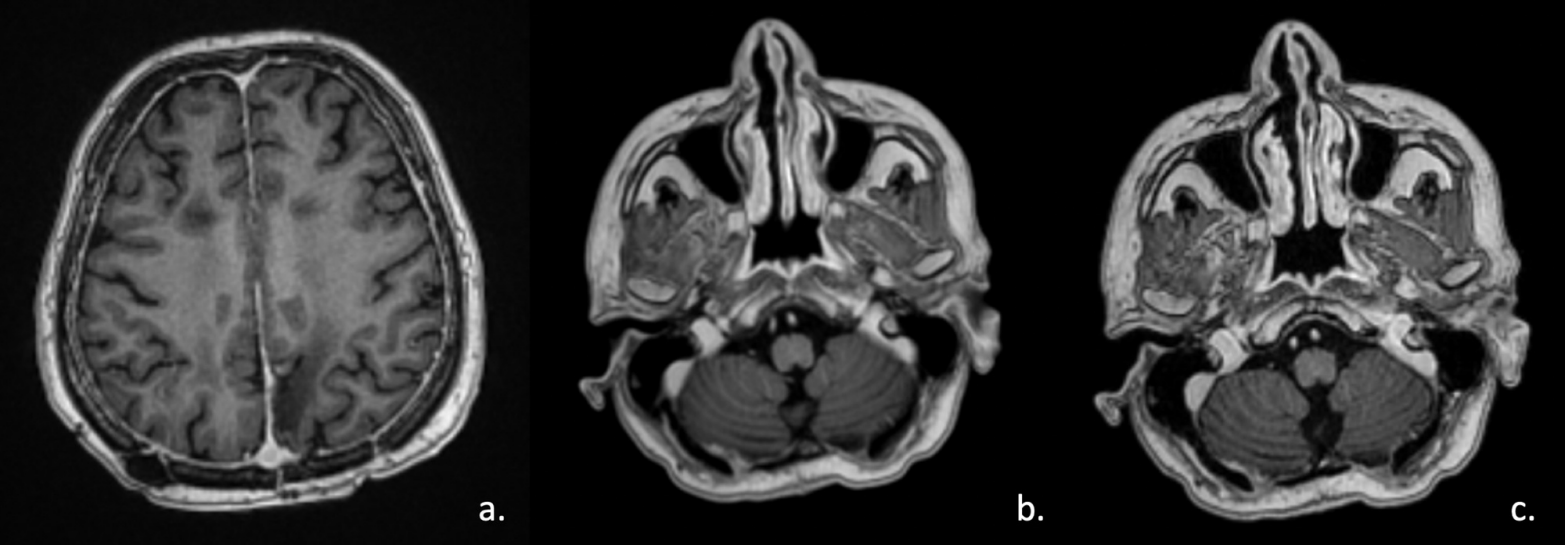
Axial contrast-enhanced MRI scans of the lesions a. In the left parietal lobe. b. In the right cerebellar hemisphere. c. In the left cerebellar hemisphere one year after the stereotactic radiosurgery treatment.

The gross tumor volumes (GTV) and the resection cavity were defined as CTV. A 2 mm margin was defined for the delineation of PTV. The patient received adjuvant three cycles of temozolomide. One year after the treatment, MRI showed stabile leptomeningeal enhancements and complete response in the left cerebellar lesion and right cerebellar lesion (Figure 4). 

**Figure 5:**
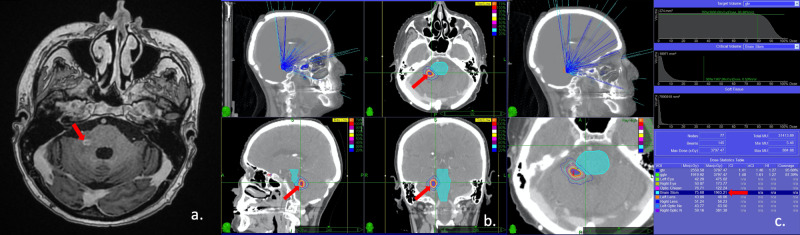
MRI of the lesion and treatment planning images a. Axial contrast-enhanced magnetic resonance imaging (MRI) scan of the new lesion in the right middle cerebellar peduncle. b. Axial, sagittal, and coronal view of the treatment planning CT scan of the lesion in the right middle cerebellar peduncle. The orange line is the prescribed isodose (79%). c. Dose-volume histogram for the lesion in the right middle cerebellar peduncle (red arrow shows the maximum dose of the brain stem).

In October 2019, a 6 mm sized new lesion was observed in the right middle cerebellar peduncle and hypofractionated SRS to a total dose of 30 Gy in five fractions was performed to the lesion using CyberKnife every other day. The dose was prescribed to the 79% isodose line normalized to the maximum dose (Figure 5).

The treatment was well-tolerated. The patient was disease-free without any treatment-related toxicity with a six-month follow-up.

## Discussion

PPT is very rare among primary brain tumors, accounting for 14%-27% of all pineal region tumors [1], and PPID accounts for approximately 10% of all PPTs [5]. According to the WHO 2016 classification, PPT is classified into pineocytoma (WHO grade I), PPID (WHO grades II-III), and pineoblastoma (WHO grade IV) [6]. Jouvet et al. proposed PPIDs less than six mitotic figures and the presence of neurofilament protein as grade II, and more than six mitotic figures without neurofilament as grade III [4]. However, due to its rarity, a clear classification has never been done. Since some tumors among the grade II-III PPT group can behave aggressively, the therapeutic options may vary widely, and the treatment mostly depends on the physician’s choice. As PPID is a rare disease, data about standard treatment are scarce.

The combination of surgery, RT, and chemotherapy may be reasonable for PPID, however, indications of adjuvant treatment depend on the physician's practice. Gross total resection is considered as the standard of care but it is not always feasible due to the depth at which the tumor is located and the tumors’ proximity to major venous structures [2]. Although minimally invasive surgical methods have been improved, the surgery may still have adverse effects and even with adequate experience, only subtotal resection is feasible in most of the cases. In adjuvant RT series, the radiosensitivity of PPTs has been described earlier [7]. In a systematic review, the addition of adjuvant RT was shown to provide a survival advantage [5]. Ito et al. reported that a complete response was achieved in cases that received RT over 50 Gy [7]. Mallick et al. reported that although there is no consensus, a dose of 50.4-54 Gy should be adequate in the adjuvant setting [5]. This review also reported that only one-third of the patients were suitable for gross total resection and due to the difficult location, another one-third of patients were managed with a biopsy only [5].

SRS has been reported as safe and effective in treating PPTs [8-14]. Most of the studies in the literature are about SRS with the Gamma Knife (Elekta, Stockholm, Sweden). However, Park et al. reported that six cases have been treated with fractionated SRS using Cyberknife and they demonstrated that the tumors responded well to hypofractionated SRS. The most commonly used fractionation scheme was 30 Gy in five daily fractions, but 36 Gy in five daily fractions was used in two patients. Only one patient who received 36 Gy in five daily fractions developed temporary memory impairment [13]. Iorio-Morin et al. reported a new onset of Parinaud’s syndrome, a new focal neurological deficit, and a worsening of hydrocephalus in their GK series [15]. In the current case, the patient tolerated the treatment well although re-irradiation was administered. SRS has several advantages, such as image-guided delivery of high-dose radiation to the target, high conformality, and steep dose fall-off, to avoid irradiating organs at risk and not requiring a rigid head frame [16]. Furthermore, hypofractionated SRS may provide additional normal tissue protection when treating tumors near functional regions [16].

The benefit of chemotherapy still remains unclear in PPID, chemotherapy should not be routinely recommended in the adjuvant setting in patients with PPID, however, in cases with recurrent disease or cerebrospinal fluid (CSF), dissemination chemotherapy can be considered [5]. There is no standard chemotherapy regimen for PPID. In our case, complete response was achieved by adding three cycles of adjuvant temozolomide after craniospinal irradiation when CSF dissemination was detected.

As late recurrences are common in this group of patients, these patients should be kept in close follow-up with contrast-enhanced brain MRI. Leptomeningeal dissemination occurs in 36% of patients with high-grade PPT and 7% of patients with low-grade PPT [17-18]. In patients with PPID, the leptomeningeal recurrence rate is only 11.8% in all reports in the literature [5]. Craniospinal irradiation is not routinely recommended, but it should be administered in case of CSF dissemination. There are only a few studies demonstrating recurrence patterns and all reported recurrences within the pineal region [19-20]. This is the first case to report recurrences in the parietal and cerebellar lobes. Since PPID is seen uncommonly, the data about the treatment of the recurrent disease is also controversial. There are a few reports about the treatment of primary disease with GK and CK [8,10,13-15]. However, only Iorio-Morin et al. reported that SRS with GK was used for three cases with recurrent PPID, and SRS doses were not specified [15]. To our knowledge, this is the first report that used hypofractionated SRS for recurrent/ metastatic PPID.

## Conclusions

Data about the optimal treatment of PPID are scarce due to its varying aggressive behavior. Combined modality treatment may be required for patients with PPID due to its high recurrence rate. This is the first case reporting reirradiation with hypofractionated SRS for multiple recurrent PPID lesions after the primary disease was controlled with the combination of surgery and conventional fractionated RT. A complete response was achieved without any treatment-related toxicity. Hypofractionated SRS can be an alternative to surgery for recurrent PPID when optimal surgery is not feasible due to its adverse effects and morbidities.
